# Long‐term continental changes in wing length, but not bill length, of a long‐distance migratory shorebird

**DOI:** 10.1002/ece3.2898

**Published:** 2017-04-04

**Authors:** David B. Lank, Cailin Xu, Brian A. Harrington, Richard I. Guy Morrison, Cheri L. Gratto‐Trevor, Peter W. Hicklin, Brett K. Sandercock, Paul Allen Smith, Eunbi Kwon, Jennie Rausch, Lisa D. Pirie Dominix, Diana J. Hamilton, Julie Paquet, Sydney E. Bliss, Sarah G. Neima, Christian Friis, Scott A. Flemming, Alexandra M. Anderson, Ronald C. Ydenberg

**Affiliations:** ^1^ Centre for Wildlife EcologySimon Fraser UniversityBurnabyBCCanada; ^2^ ManometManometMAUSA; ^3^ National Wildlife Research Centre, Environment and Climate Change CanadaCarleton UniversityOttawaONCanada; ^4^ Prairie and Northern Wildlife Research Centre, Environment and Climate Change CanadaSaskatoonSKCanada; ^5^ Canadian Wildlife Service, Environment and Climate Change CanadaSackvilleNBCanada; ^6^ Division of BiologyKansas State UniversityManhattanKSUSA; ^7^ Canadian Wildlife Service, Environment and Climate Change CanadaYellowknifeNTCanada; ^8^ Canadian Wildlife Service, Environment and Climate Change CanadaIqaluitNUCanada; ^9^ Department of BiologyMount Allison UniversitySackvilleNBCanada; ^10^ Canadian Wildlife Service, Environment and Climate Change CanadaTorontoONCanada; ^11^ Environmental and Life SciencesTrent UniversityPeterboroughONCanada; ^12^ Present address: Department of Fish and Wildlife ConservationVirginia TechBlacksburgVAUSA

**Keywords:** allometry, *Calidris pusilla*, environmental change, phenotypical change, predation risk, semipalmated sandpiper

## Abstract

We compiled a >50‐year record of morphometrics for semipalmated sandpipers (*Calidris pusilla*), a shorebird species with a Nearctic breeding distribution and intercontinental migration to South America. Our data included >57,000 individuals captured 1972–2015 at five breeding locations and three major stopover sites, plus 139 museum specimens collected in earlier decades. Wing length increased by ca. 1.5 mm (>1%) prior to 1980, followed by a decrease of 3.85 mm (nearly 4%) over the subsequent 35 years. This can account for previously reported changes in metrics at a migratory stopover site from 1985 to 2006. Wing length decreased at a rate of 1,098 darwins, or 0.176 haldanes, within the ranges of other field studies of phenotypic change. Bill length, in contrast, showed no consistent change over the full period of our study. Decreased body size as a universal response of animal populations to climate warming, and several other potential mechanisms, are unable to account for the increasing and decreasing wing length pattern observed. We propose that the post‐WWII near‐extirpation of falcon populations and their post‐1973 recovery driven by the widespread use and subsequent limitation on DDT in North America selected initially for greater flight efficiency and latterly for greater agility. This predation danger hypothesis accounts for many features of the morphometric data and deserves further investigation in this and other species.

## 1 Introduction

The morphometry of populations shifts constantly in response to environmental changes in biological and physical factors. The colors and markings of male guppies *Poecilia reticulata* quickly evolved lower intensity when predators were experimentally introduced (Endler, 1980). Beak sizes of Galápagos finch species (*Geospiza* spp.*)* have undergone rapid and fluctuating changes in response to severe natural selection on their performance under varying abundance, size, and hardness of seeds produced in response to variation in rainfall (Grant, 1986). The wings of forest birds in eastern North America have become more pointed in boreal regions and less pointed in temperate regions over the past century, which Desrochers (2010) interpreted as resulting from selection pressure arising from landscape changes, specifically deforestation in boreal regions (favoring more flight) and afforestation in temperate regions (less flight). A 20‐year decline in wing size observed in cliff swallows (*Petrochelidon pyrrhonota*) in Nebraska was attributed to mortality from automobile collisions favoring increased agility (Brown & Brown, 2013). Shorter, more convex, and less pointed wings increase lift and hence take‐off speed, and improve flight agility, both important traits for evading predators (Swaddle & Lockwood, 1998; Burns & Ydenberg, 2002; Burns, 2003). However, they are less energetically efficient for sustained flight due to their greater drag (Savile, 1956; Rayner, 1988; Norberg, 1990; Hedenström & Møller, 1992; Pennycuick, Fuller, Oar, & Kirkpatrick, 1994; Vágási et al., 2016).

Morphometric change may result from natural or sexual selection, as in all of the above examples, but can also be due to phenotypic plasticity (Teplitsky, Mills, Alho, Yarrall, & Merilä, 2008). Declines in overall adult body size in several European passerine bird populations appear to be environmental effects on phenotype and were attributed to climate warming causing an increasing temporal mismatch between the peak in food abundance and the chick‐rearing period (Husby, Hille, & Visser, 2011). Plasticity was also invoked to account for much of the changes in body proportions of arctic‐breeding red knots (*Calidris canutus*, van Gils et al., 2016).

Whether by microevolution, phenotypic plasticity, or some combination of both processes, several authors have proposed that a decline in body size is a widespread general response to climate warming (Rode, Amstrup, & Regehr, 2010; Gardner, Peters, Kearney, Joseph, & Heinsohn, 2011; Sheridan & Bickford, 2011; Baudron, Needle, Rijnsdorp, & Marshall, 2014). Empirical papers have documented heterogeneity in the magnitude and direction of size responses of many taxa (Yom‐Tov, Yom‐Tov, Wright, Thorne, & Du Feu, 2006) and call for both empirical and theoretical studies to better understand the underlying mechanisms and physiological consequences of body size shifts (McNamara, Higginson, & Verhulst, 2016). Such studies contribute to our understanding of the extent to which, and how rapidly, species may respond to macroenvironmental changes (Botero, Dor, McCain, & Safran, 2014).

This study examines long‐term variation in two measures of body size in a long‐distance migratory shorebird, the semipalmated sandpiper (*Calidris pusilla*). The species breeds across arctic North America and winters along coastlines of Central and South America (Hicklin & Gratto‐Trevor, 2010). Our investigation was stimulated by reports that semipalmated sandpipers captured at Johnson's Mills in the Bay of Fundy in the early 1980s had substantially longer wings and slightly longer bills than those caught during the late 1990s and early 2000s (Hicklin & Chardine, 2012). The species has a marked geographic cline in size across the breeding range, with bills and wings shorter in the west (Harrington & Morrison, 1979; Gratto‐Trevor et al., 2012). Western and some central Arctic breeders migrate southward through central North America (Gratto‐Trevor et al., 2012). The remaining central Arctic breeders, and all eastern Arctic birds, migrate south via the Atlantic coast, particularly the Bay of Fundy. Hicklin and Chardine (2012) interpreted the shorter metrics they reported around 2000 as support for the hypothesis that eastern, long‐billed populations had recently undergone large and disproportionate population reductions, resulting in relatively lower usage of this migratory site than populations from central breeding areas. Surveys in North America (Morrison et al., 2001, 2006) and South America (Ottema & Spaans, 2008; Morrison et al., 2012) have reported strong declines, thought to represent primarily eastern populations (Brown et al., 2017; but see Andres et al., 2012).

Interpreting changes in morphometrics of birds captured at migratory stopover sites is complicated because changes in the metrics can occur in several ways related to sample biases, even in the absence of any real phenotypic changes. If body size differs among breeding areas, changing representation bias from different areas of origin would alter metrics (Hicklin & Chardine, 2012). If the sexes differ in size or passage time, a change in sex ratio or the timing of sampling relative to this progression could also alter population metrics. Last, changes in size distribution at a stopover site could result from mass or morphology‐dependent shifts in habitat use (e.g., mass‐dependence: Ydenberg et al., 2002; Ydenberg, Butler, Lank, Smith, & Ireland, 2004). Any or all of these processes could apply to semipalmated sandpipers.

Identifying phenotypic change in the morphometrics of breeding populations of semipalmated sandpipers is more straightforward because none of these complications is a factor. We therefore undertook a range‐wide investigation of historical changes in wing and bill morphology in this species at breeding and stopover sites, to determine the extent to which the large changes reported represented true morphological changes versus changes in migratory demographics and timing. Specifically, we tested the possibility that phenotypic change on the breeding range, rather than or in addition to changes in migratory population structure, could account for changes reported at stopover sites (Hicklin & Chardine, 2012). We compiled morphometric data measured on breeding populations of semipalmated sandpipers to help interpret the long‐term changes recorded at migratory stopover sites, including data from two additional migratory sites to help assess whether the morphometric changes reported from the Bay of Fundy also occurred elsewhere.

## 2 Methods

We assembled field measurements of bill and wing length from >57,000 semipalmated sandpipers captured as live adults between 1972 and 2015 at five breeding sites across the Arctic and at three major southbound migratory stopover sites. Morphometric data originate from both published and unpublished reports. The locations, years, sample sizes, annual means and standard errors, references to methods and to persons supervising data collection are summarized in Table 1. Data from these “live birds” were compared with measures from 139 museum specimens of adults collected earlier, mostly during the 1950s and 1960s, with a few specimens dating back to the early twentieth century (Harrington & Morrison, 1979). To compare metrics from museum metrics with those from live birds, we adjusted for shrinkage by increasing the measured culmen length of each museum specimen by 1% (Engelmoer & Roselaar, 1998) and wing length by 2% (Prater, Marchant, & Vuorinen, 1977; Harrington & Morrison, 1979). Museum specimens were sexed by gonadal inspection.

**Table 1:** Summary table of the morphometric data (wing length, exposed culmen length) of adult semipalmated sandpipers

Location and References	Year	*N* wings or (wing, culmen)	Wing (mean ± *SE*)	Culmen (mean ± *SE*)	Measured/orsupervised by
*Western Breeding Region*					
Harrington & Morrison, 1979	Historical	Simulated (32,21)	96.66 ± 2.48^a^	18.27 ± 1.02^a^	RIGM, BH
Nome, AK^b^, 64.333°N, 164.933°W Sandercock, 1998; Sandercock et al. 2000; BKS, EK unpublished^c^	1993	194	97.94 ± 0.19	17.96 ± 0.08	BKS
	1994	131	97.45 ± 0.24	17.92 ± 0.10	BKS
	1995	118	98.18 ± 0.25	17.64 ± 0.12	BKS
	1996	46	97.80 ± 0.34	17.76 ± 0.19	BKS
	1998	35	96.90 ± 0.45	17.24 ± 0.28	DS
	1999	20	98.60 ± 0.58	17.90 ± 0.16	DBL
	2010	32	96.91 ± 0.45	18.55 ± 0.23	DBL
	2011	90	95.02 ± 0.28	18.39 ± 0.18	BKS, EK
	2012	69	94.74 ± 0.36	18.11 ± 0.13	BKS, EK
	2013	58	95.77 ± 0.33	18.24 ± 0.15	BKS, EK
	2014	32	95.64 ± 0.37	17.94 ± 0.18	BKS, EK
*Central Breeding Region*					
Harrington & Morrison, 1979	Historical	Simulated (47,47)	97.4 ± 3.03^a^	18.86 ± 1.18^a^	RIGM, BH
Mackenzie Delta, NT 69.333°N, 135.500°W CLGT, JR, LPD unpublished^c^	1991	3	99.67 ± 0.88	18.53 ± 0.44	CLGT
	1992	8	98.50 ± 087	18.62 ± 0.47	CLGT
	1994	11	100.00 ± 0.77	18.90 ± 0.44	CLGT
	2010	9	95.89 ± 0.73	17.90 ± 0.44	JR and LPD
	2011	14	96.70 ± 0.98	17.99 ± 0.49	JR and LPD
	2012	16	94.38 ± 0.61	18.48 ± 0.31	JR and LPD
	2013	28	95.25 ± 0.56	18.14 ± 0.32	JR and LPD
	2014	27	97.80 ± 1.13	18.56 ± 0.21	JR and LPD
Rasmussen, NU 68.667°N, 93.000°W CLGT, unpublished	1994	11	100.00 ± 0.77	18.90 ± 0.44	CGLT
*Eastern Breeding Region*					
Harrington & Morrison, 1979	Historical	Simulated (60,60)	99.34 ± 2.92^a^	20.65 ± 1.25^a^	RIGM
La Pérouse Bay, MB 54.400°N, 94.400°W Gratto, Cooke, & Morrison, 1983	1980	40	101.10 ± 0.36	20.64 ± 0.18	CLGT
	1981	52	100.25 ± 0.34	20.42 ± 0.18	CLGT
	1982	60	100.52 ± 0.31	20.57 ± 0.16	CLGT
	1983	55	100.47 ± 0.32	20.83 ± 0.16	CLGT
	1984	54	100.15 ± 0.30	20.58 ± 0.15	CLGT
	1985	66	100.20 ± 0.27	20.59 ± 0.13	CLGT
	1986	33	100.30 ± 0.44	20.58 ± 0.21	CLGT
	1987	28	100.29 ± 0.45	20.29 ± 0.22	CLGT
Coats Island, NU 62.852°N, 82.485°W PAS, SF unpublished^c^	2004	35	95.69 ± 0.40	19.95 ± 0.25	PAS
	2005	15	98.27 ± 0.81	20.59 ± 0.33	PAS
	2013	34	97.91 ± 0.56	19.68 ± 0.21	PAS
	2014	28	99.50 ± 0.36	19.88 ± 0.27	SGN
	2015	48	97.31 ± 0.41	20.04 ± 0.21	PAS, SAF
Stopover site, Manomet, MA^d^ 41.919°N, 70.541°W Harrington & Morrison, 1979	1972	1,118	92.33 ± 0.08	19.89 ± 0.05	BH + PD
	1973	682	93.04 ± 0.10	20.41 ± 0.06	BH + PD
	1976	366	93.41 ± 0.12	20.45 ± 0.08	BH + PD
	1977	334	93.54 ± 0.16	20.12 ± 0.08	BH + PD
	1978	456	92.59 ± 0.11	20.20 ± 0.07	BH + PD
	1979	542	92.78 ± 0.11	19.92 ± 0.06	BH + PD
	1985	607	94.53 ± 0.10	20.28 ± 0.06^d^	BH + PD
	1986^e^	25	95.12 ± 0.52	19.25 ± 0.30^d^	BH + PD
	1987	200	92.90 ± 0.16	20.32 ± 0.10^d^	BH + PD
	1988	170	92.76 ± 0.17	19.92 ± 0.14^d^	BH + PD
	1989	122	93.72 ± 0.26	20.11 ± 0.15^d^	BH + PD
	1990	255	93.60 ± 0.16	19.75 ± 0.11^d^	BH + PD
	1991^e^	58	93.98 ± 0.36	18.89 ± 0.21^d^	BH + PD
	1993	286	94.28 ± 0.15	20.56 ± 0.08^d^	BH + PD
	1994	142	94.11 ± 0.21	19.71 ± 0.13^d^	BH + PD
	1995^e^	54	93.56 ± 0.37	20.65 ± 0.20^d^	BH + PD
Stopover site, North Point, James Bay, ON 51.484°N, 80.450°W Harrington & Morrison, 1979; RIGM, CLGT, CF, AMA unpublished	1975	2,202	98.75 ± 0.05	19.12 ± 0.03	RIGM, CLGT
	1976	6,013	99.87 ± 0.03	19.42 ± 0.02	RIGM, CLGT
	1977	5,299	99.27 ± 0.03	19.48 ± 0.02	RIGM, CLGT
	1978	5,047	99.92 ± 0.03	19.63 ± 0.02	RIGM, CLGT
	1979	2,152	100.23 ± 0.05	19.52 ± 0.03	RIGM, CLGT
	1980	1,408	100.12 ± 0.07	19.48 ± 0.03	RIGM, CLGT
	1981	1,357	99.68 ± 0.07	19.90 ± 0.03	RIGM, CLGT
	1982	1,690	99.26 ± 0.06	19.68 ± 0.03	RIGM, CLGT
	2014	227	97.10 ± 0.17	19.19 ± 0.09	CF
	2015	(104, 52)	98.36 ± 0.24	19.40 ± 0.19	CF, AMA
Stopover site, Bay of Fundy, NB 45.829°N, 64.509°W Hicklin & Chardine, 2012; PH, JP, P. Donahue, N. Garrity pers. comm., Bliss, 2015	1981	1,290	100.50 ± 0.08	20.22 ± 0.04	PH
	1982	1,225	99.39 ± 0.07	20.09 ± 0.04	PH
	1984^e^	88	99.73 ± 0.29	20.85 ± 0.15	PH
	1986	1,811	97.66 ± 0.06	20.18 ± 0.04	PH
	1987	1,335	98.97 ± 0.08	20.23 ± 0.04	PH
	1989	272	97.34 ± 0.08	20.02 ± 0.09	PH
	1997^f^	1,776	97.02 ± 0.07	19.85 ± 0.03	PD, 8 others
	1998^f^	1,304	93.81 ± 0.07	19.90 ± 0.04	PD, 4 others
	1999^f^	1,592	95.19 ± 0.06	19.68 ± 0.03	PD, 1 other
	2000^f^	885	95.40 ± 0.08	19.38 ± 0.05	PD
	2001^f^	1,878	93.34 ± 0.07	19.54 ± 0.03	PD
	2002^f^	1,902	92.25 ± 0.06	19.83 ± 0.03	PD
	2003^f^	858	92.66 ± 0.09	19.34 ± 0.04	PD
	2004^f^	739	93.90 ± 0.09	19.19 ± 0.05	NM, NG
	2005^f^	213	93.85 ± 0.17	19.58 ± 0.10	NG
	2006^f^	(75, 1,030)	98.11 ± 0.32	19.75 ± 0.05	–
	2012	717	97.65 ± 0.09	19.68 ± 0.05	CLGT, JP
	2013	1,153	97.94 ± 0.08	20.12 ± 0.04	CLGT, JP
	2014	1,179	98.21 ± 0.08	20.29 ± 0.04	CLGT, JP, DJH

Wing lengths are flattened and straightened lengths, except as footnoted. The annual mean and standard error, and the identity of the banding supervisor are given for each of five breeding and three stopover locations. Sample sizes (*n*) are given for wing measurements; sample sizes for culmen are within 1% of the matching wing tally, except as noted with double entries: (wing, culmen). “Historical” wing and culmen lengths were simulated as described in the text, *n* in this case refers to the number of museum specimens originally measured, and standard deviations rather than *SE*s are listed. Bander/supervisor initials: AMA, Alexandra M. Anderson; BH, Brian Harrington; BKS, Brett K. Sandercock; CF, Christian Friis; CLGT, Cheri L. Gratto‐Trevor; DBL, David B. Lank; DJH, Diana J. Hamilton; DS, Doug Schamel; EK, Eunbi Kwon; JP, Julie Paquet; JR, Jennie Rausch; NG, Neville Garrity; NM, Nic McLellan; PAS, Paul A. Smith; PD, Paul Donahue; PH, Peter Hicklin; RIGM, R.I.G. Morrison, SAF, Scott A. Flemming; SGN, Sarah G. Neima.

a *SD* presented for simulated data.

b Includes only birds caught at nests; excludes 66 additional adults trapped in mist nests 1993–1995 after nests hatched. The late mist netted population had shorter wings (wings: nest trap: *N* = 447, mean = 97.8, mist nets: *N* = 66 mean = 95.7, *t*_[553]_ = 6.08, *p *<* *.0001), probably due to a more male‐biased sex ratio, as females depart breeding grounds earlier than males (Ashkenazie & Safriel, 1979; Gratto‐Trevor, 1991).

c Data gathered 2010–2014 in coordination with the Arctic Shorebird Demographics Network (Brown, Gates, & Liebezeit, 2014).

d All wing data natural wing chord. Culmen length from 1985 to 1995 were adjusted from a “narina” bill measurement taken in those years, using the regression equation: culmen = 1.08 + 1.04*narina, based on 168 birds measured both ways in 1988 (*r* ^2^ = 0.96, *F*_[1,167]_ = 3885.3, *p *<* *.0001).

e Data from Manomet in 1986, 1991, and 1995, and from the Bay of Fundy in 1984 and 2006 (wings only, *n* = 75 from 1 day) were excluded from trend analysis due to limited temporal capture effort those years and therefore probable sex bias.

f Wing data from the Bay of Fundy from 1979 to 2006 were excluded from trend analyses due to annual differences in measurement techniques (see text).

Live birds from breeding grounds were trapped on nests as routine parts of breeding biology studies (Table 1). To account for the known geographic cline in body size of semipalmated sandpipers across their Arctic breeding range (Harrington & Morrison, 1979), each record was assigned to “western” (Alaska); “central,” including western Nunavut, Banks Island, Kitikmeot (formerly Mackenzie) and Kivallig (formerly Keewatin) Districts of the Northwest Territories; or “eastern” regions (Baffin Island, Belcher Island/eastern Hudson Bay), using the divisions portrayed in Gratto‐Trevor et al. (2012). Adult semipalmated sandpipers at migration stopover sites were captured in mist nets or, after 1986 at the Bay of Fundy, primarily with Fundy pull traps at roosting sites (Hicklin, Hounsell, & Finney, 1989).

For each bird, bill length was taken as the exposed culmen, measured with calipers to the nearest 0.1 mm. Wing measurements were taken using wing rulers; precision was 1.0 mm at most sites, but 0.5 mm at some. At most sites in most years, “flattened” wing length, from a bent “elbow” (radius/ulna to carpus‐metacarpus joint) to primary tip, was measured, which has become the worldwide standard for shorebirds (Prater et al., 1977). At Manomet (1972–1995), researchers measured “natural” wing chord, which in semipalmated sandpipers is 2–4 mm (1.5%–3%) shorter than flattened chord, varying with individual researcher (D. B. Lank, C. Friis, S. E. Bliss, unpublished data). At the Bay of Fundy stopover site, there were annual and observer differences in wing measurement techniques between 1997 and 2006 (P. Hicklin, J. Paquet, P. Donahue, N. Garrity, pers. commun.; Table 1). We therefore treat the wing lengths from Manomet, and the Bay of Fundy 1997–2006, separately in our analyses and interpretations.

### 2.1 Data analysis

**Figure 1:**
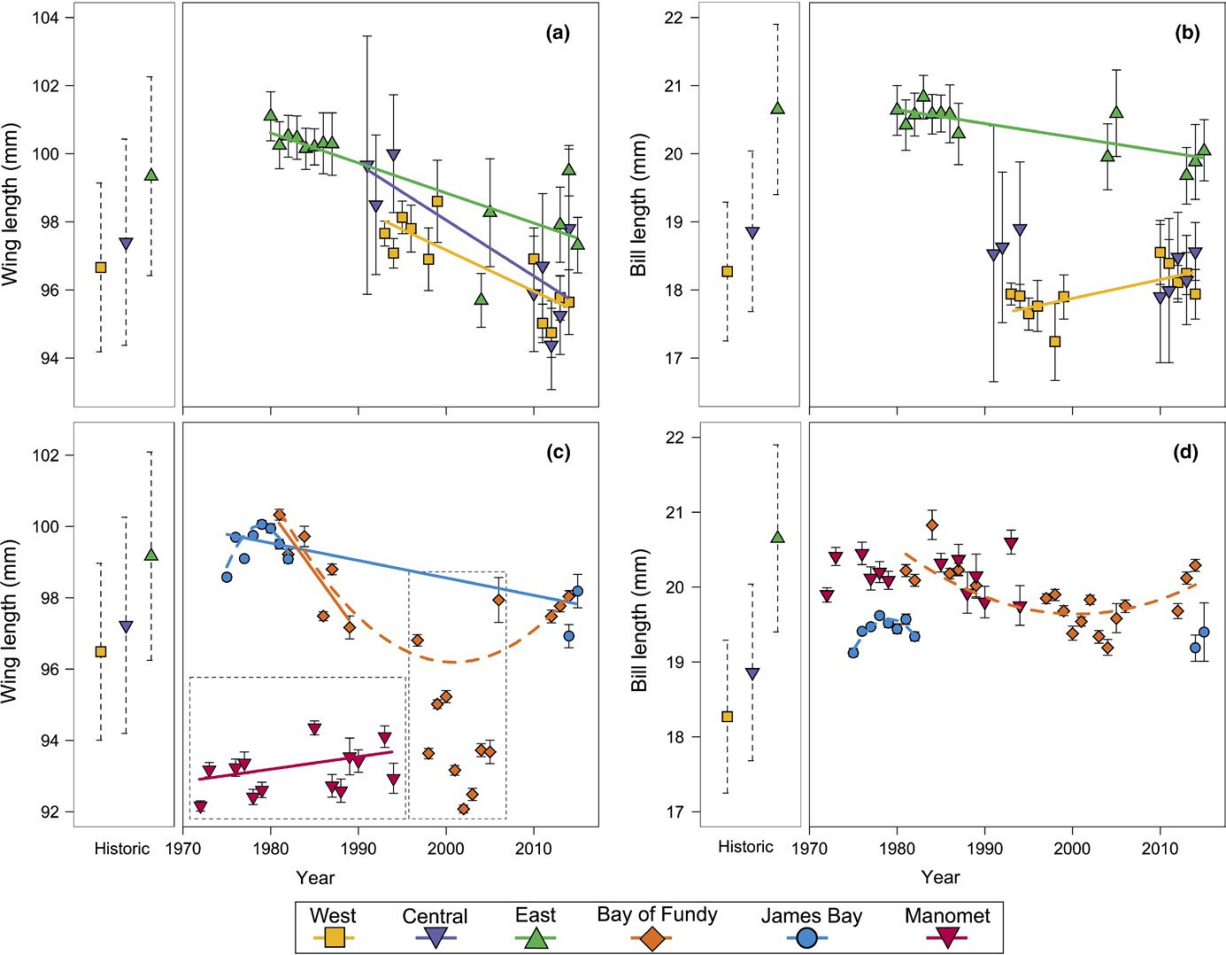
Five decades of annual mean wing (left panels: a, c) and bill lengths (right panels: b, d) of adult semipalmated sandpipers, measured at breeding (upper panels: a, b) and migratory stopover sites (lower panels: c, d). The measures from live birds are plotted as annual means in mm with 95% CIs. The left portion of each panel displays historical regional wing and culmen distributions (mean ± *SD*), estimated based on pre‐1970 museum specimens, as described in the text. Breeding sites are aggregated into three breeding regions (west, central, east; see text). Stopover measures were made during southward migration at three major stopover sites (James Bay, ON; Bay of Fundy, NB; Manomet, MA). Lines indicate statistically significant linear (solid) or quadratic (dashed) trends in annual mean values for individual breeding regions or stopover sites. A few points from James Bay and Manomet stopover sites were excluded from trend calculations because sampling did not occur throughout the season (see text, Table 1). Wing measurements are flattened chords, except for points within the dashed boxes, which are natural wing chords recorded at Manomet, or of annually variable methodology at the Bay of Fundy 1997–2006 (see text, Table 1). Bill lengths were measured as exposed culmen (Table 1)

Our analytical approach compares estimated metrics of samples or populations with balanced sex ratios. Sex‐specific comparisons of live birds are difficult because semipalmated sandpipers have female‐biased sexual size dimorphism (Harrington & Taylor, 1982), but cannot be reliably sexed in the field; ~20%–30% of individuals remain ambiguous even with information from 11 skeletal variables (Cartar, 1984). To estimate size distributions of “historical” wing and bill lengths for each region, we first generated normal size distributions from sex‐specific means and standard deviations of the pooled museum specimens within each region (Harrington & Morrison, 1979; their Table 2). We then randomly drew 1,000 males and 1,000 females from the simulated regional distributions and pooled the sexes to estimate a regional population mean and standard deviation (*SD*). These values are displayed in the left portion of each panel in Figure 1.

**Table 2:** Wing and culmen length in the simulated historical distributions (see text, Table 1, Figure 1) compared with those of live birds from the earliest breeding studies in each region (see text)

Region	Years live birds measured	Wing				Culmen			
		*N* random, live	Mean difference (mm)	*t*	*p*	*N* random, live	Mean difference (mm)	*t*	*p*
West	1993–1999	32, 555	+0.95	2.22	.10	32, 559	−1.09	**−**1.82	.12
Central	1991–1994	47, 22	+2.00	2.82	.02	46, 21	−0.13	**−**0.33	.68
East	1980–1987	49, 388	+1.03	2.80	.04	70, 388	−1.24	**−**0.25	.56

Positive differences indicate that the historical mean is shorter. The differences in *t* and *p* values reported are the means of 30 two‐sample *t* tests, in which random samples with size equivalent to the number of museum specimens originally measured were drawn from the simulated historical regional distributions, and compared against the corresponding live distributions.

We compared baseline historical distributions with the earliest morphometrics from live breeding birds that were available from each region (eastern: 8 years, 1980–1987; central: 3 years between 1991 and 1994; western: 6 years, 1993–1998; Table 1 and Figure 1). In breeding studies, both members of this socially monogamous species with biparental incubation were usually trapped at the nest, and these field samples were therefore well balanced by sex. We tested measurements from all sites pooled within each region against random samples drawn from the simulated historical distributions. To make comparisons with appropriate statistical power, we drew random samples from the historical distributions, with replacement, each with *N* equal to the number of museum specimens originally measured for that region and metric (Harrington & Morrison, 1979; their Table 2). We then compared the live versus historical distributions with two‐sample *t* tests. We report the mean *t*‐values and probabilities from tests against 30 random samples drawn from each region's historical distribution (Table 2).

We contrasted measurements made during the 1980s and 1990s with available measurements made in 2004 and later, with most made after 2010 (Table 1), using data pooled within “early” versus “late” periods and two‐sample *t* tests. To test for trends across years, our unit of analysis was the annual mean per region or migration site, rather than the original data. This allows us to present tests of comparable statistical power despite large differences in sample sizes among sites and years. When four or more years of data were available, we tested for temporal trends that were linear (year) or quadratic (year + year^2^) with standard least‐squares regression; the data were too sparse to meaningfully examine fits to higher polynomials. We compared annual rates of change in metrics among regions by testing for interactions (region*year) with analysis of covariance, although inference was limited due to little overlap of time periods with data among regions. Because differential migration can produce seasonal biases in sex ratio (Hicklin & Chardine, 2012), we excluded from trend analyses site‐years when birds were sampled for limited time periods (Bay of Fundy: 1984, 2006 for wing lengths only; Manomet: 1986, 1991, 1995). An alternative approach using least‐squared annual means corrected for date of capture to adjust for sex ratio effects at migration sites, assuming common seasonal slopes over years (Hicklin & Chardine, 2012), produced results similar to those presented here.

For comparison with other studies, we estimate rates of phenotypic change in darwins and haldanes (Hendry & Kinnison, 1999). Almost all wing and bill metrics were taken on the same individuals, allowing us to calculate the phenotypic covariances of samples from sites to estimate what fraction of change might be attributed to a change in one metric driving change on the other and how these relationships might change over time. We present these as slopes of bill versus wing length. We used SAS 9.4 for data management and statistical calculations.

## 3 Results

The well‐known longitudinal clines in bill and wing sizes of semipalmated sandpipers are evident in the regional historical distributions portrayed on the left side of each panel in Figure 1. Mean bill length in the eastern portion of the breeding range averaged ~2.4 mm (13%) longer and mean wing length was ~2.7 mm (3%) longer than in the west, with intermediate sizes in the central region. Although the absolute values change, the differences between the regions were maintained throughout the entire time period (Figure 1a,b).

Individuals from different breeding regions mingle at migratory stopover sites and mean sizes were thus expected to be intermediate to sizes of their breeding regions of origin. This prediction was met for culmen lengths (Figure 1d); means at the Bay of Fundy and at Manomet fell between those of eastern and central populations. Bill lengths at James Bay were also intermediate to eastern and central populations and were slightly shorter than those measured at Fundy, also as expected if James Bay included a higher proportion of smaller western arctic birds. Annual mean wing length values for James Bay and the Bay of Fundy (for years with comparable measurement techniques: 1981–1989 and 2012–2015) fell between eastern and central populations, although James Bay birds were not obviously shorter than contemporaneous measurements at the Bay of Fundy, as might have been expected.

Comparisons with the historical distributions indicate that wing length increased in the decades prior to 1980, when the first breeding site studies began. Mean wing lengths during the earliest measures of live birds in each region were longer than their corresponding historical means by +0.95 mm, +2.00 mm, and +0.97 mm, in western, central, and eastern regions, respectively. The differences were unlikely to have arisen by chance (*p *=* *.04, .02, and .10, respectively; Table 2). In contrast, bill lengths from these years showed no significant differences from corresponding historical means in any region, and are if anything shorter by 1.09, 0.13, and 1.24 mm in western, central, and eastern regions, respectively (*p *=* *.12, .68 and .56, respectively; Table 2).

Within each region, wing lengths measured on live birds at breeding sites were longest in the earlier years and decline thereafter. Thus, the period of wing length increase terminated before or as these studies began. At the James Bay stopover site, a quadratic model fits the 1975–1982 data, with a 1980 peak length (annual mean wing = 309,924 + 313.33*year − 0.07917 year^2^, *F*_[1,5]_ = 9.52, *p *=* *.027). As several thousand birds were captured at this site annually, the annual estimates themselves have narrow confidence limits, and this curve provides solid support for a 1980 peak in wing length at this location.

After 1980, mean wing length decreased on breeding sites by nearly 4% over the 35‐year period ending in 2015 (Table 3, Figure 1). Analysis of covariance shows no significant regional differences in rate (region*year interaction, *F*_[2,26]_ = 1.74, *p* = .19), although the power to detect differences was limited due to little overlap of time periods among regional data; in the reduced model, annual mean wing length varies with year and region (*F*_[3,28]_= 28.52, *p *<* *.0001). The estimated common annual wing length decline was −0.11 ± 0.02 mm/year; thus by 2015, wings across the breeding range were ~3.85 mm shorter than their peak in 1980. At the James Bay stopover site, linear regression between the early and later (2014–2015) years estimates a similar slope of −0.079 mm/year (Table 3, Figure 1c). In time period contrasts, birds captured at James Bay in 2014–2015 had mean wing length 2.16 ± 0.14 mm shorter than the mean of the pooled distribution from 1975 to 1982 (1975–1982: mean = 99.67 ± 0.02; 2014–2015: mean = 97.49 ± 0.14; *t*_[25 168,332]_ = 15.60, *p *<* *.0001). In evolutionary terms, the 3.85‐mm change on the breeding grounds corresponds to a rate of change of 1098 darwins or 0.176 haldanes (based on a wing size standard deviation of 2.916 calculated by pooling breeding populations and years, and a generation time of 4.67 years, calculated by BKS from data in Hitchcock & Gratto‐Trevor, 1997).

**Table 3:** Regression coefficients of linear models of change in mean annual morphometrics of adult semipalmated sandpipers on breeding grounds and at major southward migration stopover sites

Location	Years	Wing				Culmen			
		Slope	*SE*	*t*	*p*	Slope	*SE*	*t*	*p*
Breeding									
Western	1993–2014	**−0.131**	0.027	**−**4.87	**.001**	**0.026**	0.011	2.42	**.038**
Central	1991–2014	**−0.164**	0.047	**−**3.51	**.013**	**−**0.021	0.010	**−**1.91	*.100*
Eastern	1980–2014	**−0.089**	0.022	**−**4.11	**.002**	**−0.021**	0.004	**−**4.7	**.001**
Migration									
Manomet	1972–1995	**0.055**	0.022	2.52	**0.028**	**−**.007	0.011	**−**0.67	0.518
James Bay	1975–1982	Quadratic fit see text				Quadratic fit see text			
James Bay	1975–1982, 2014	**−0.067**	0.017	**−**3.88	**.006**	**−**0.006	0.005	**−**1.21	.266
Bay of Fundy	1981–1989	**−**0.331	0.110	**−**3.01	*.057*	**−**0.009	0.014	**−**0.65	.563
Bay of Fundy	1998–2005	**−**0.184	0.167	**−**1.11	.311	**−**0.027	0.025	**−**1.09	.310
Bay of Fundy	1998–2014^a^	Quadratic fit see text				Quadratic fit see text			

Sample sizes shown in Table 1; *p *<* *.05 in bold, ≤.10 in italics.

a Excluding 1998–2005 for wings.

Wing length data from the Bay of Fundy were analyzed separately during three time periods (see section 2, Table 3, Figure 1). Flattened wing lengths, all measured by PH (Table 1), decreased steeply throughout the 1980s, shortening by 0.33 ± 0.11 mm/year. There was no trend between 1995 and 2005, although extensive annual variation, probably due in part to measurement technique differences among those years (see section 2), hinders potential trend detection. A regression of all the flattened wing chord means (1981–1989 and 2012–2014) did not support a long‐term linear trend (*p* = .18), but a quadratic fit was significant, driven by the steep initial decline and subsequent leveling off (Figure 1a, annual mean wing length = 43,200 − 43.10*year + 0.01*year^2^, *F* = 13.46_[1,5]_, *p* = .014). Visual comparison of the decrease in length between the early 1980s and 2012–2014 shows an overall rate of change comparable to those observed at James Bay and on the breeding grounds (Figure 1a,c).

Bill lengths present a less coherent picture, with no overall directional changes over the approximately 70 years considered (Table 3, Figure 1b,d). As noted above, historical bill lengths did not differ from the earliest breeding site measurements. Within the breeding site data, a significant interaction between region and year (*F*_[2,26]_ = 9.53, *p *<* *.001) indicated increases in western bill length (0.26 mm/year, *p* = .04) but decreases in the central (−0.21 mm/year, *p* = .10) and eastern (−0.21 mm/year, *p *<* *.001) regions, both changes of about 1%. Measures made at stopover sites also present a mixed picture. At James Bay, the pattern of bill length change paralleled long‐term changes in wing length, with a significant quadratic relationship from 1975 to 1982 (annual mean bill = −98,592 + 99.66*year − 0.03*year^2^, *F* = 14.90_[1,5]_, *p* = .012) and shorter bills in 2014–2015 than earlier (linear slope = −0.003 mm/year, difference = −0.23 ± 0.09 mm; 1975–82: mean = 19.46 ± 0.01; 2014–2015: mean = 19.23 ± 0.08; *t*_[25 168,332]_ = 2.92, *p* = .004). For the Bay of Fundy, modeling of all years’ bill length means showed support for a quadratic, but not a linear fit (mean bill length = 8510.04 − 8.49* year + 0.002*year^2^, *F* = 13.28_[1,15]_, *p* = .002), similar to the pattern found for wings (Figure 1d). Thus after the decline in the 1990s found by Hicklin and Chardine (2012), in recent years, mean bill lengths returned to historical values (Figure 1d, Bliss 2015).

Data from the migratory stopover site at Manomet, Massachusetts, were available from the mid‐1970s through 1995. Despite large samples in most years, wing length means fluctuated considerably, but on average increased by ~1.3 mm. There was no temporal trend in bill length (Tables 1 and 3, Figure 1c,d).

There was substantial phenotypic covariance between the two body size metrics (Table 4). Regressions of bill length on flattened wing lengths produce slopes ranging from 0.109 to 0.336 mm bill/mm wing for different samples with an overall mean slope of 0.223. The mean covariance predicts that the 3.85‐mm decrease in wing size observed over 35 years would be matched with a decrease of ca. 0.84 mm in bill length (Table 4). The value reasonably matches the observed decrease for the central and eastern breeding samples (−0.021*35 years = −0.74 mm), but mismatches the increase in bill length in the west (+0.91 mm). Among sampling sites, the slope of bill as a function of wing length decreases over time (regression of data in Table 4, weighted by sample size, with points for each site and time period centered on time periods measured: slope = 11.38 − 0.0056*year; *F*_1,6_ = 16.03, *p* = .01), reflecting the maintenance of longer bill lengths despite shortening of wings. Thus, body shape has changed over the period.

**Table 4:** Phenotypic covariances between culmen and wing measurements of individual adult semipalmated sandpipers, and the regression slopes of culmen versus wing in the breeding regions (west, central, east) or during fall migration at the Bay of Fundy and James Bay

Location	Time period	*N*	Covariance	Slope (Culmen~Wing)
James Bay	1975–1982	25168	1.661	0.257
East	1980–1987	388	1.609	0.299
Bay of Fundy	1981–1989	5998	1.725	0.207
Central	1991–1992, 1994	22	1.907	0.325
West	1993–1996	555	1.085	0.143
East	2004–2005	50	2.747	0.336
West	2011–2012	159	0.885	0.111
East	2013	34	1.177	0.109
Mean slope				0.223

All slopes significant at *p *<* *.0001.

## 4 Discussion

Semipalmated sandpipers have undergone two large changes in wing length over the past 50–70 years. Wing lengths appear to have been increasing prior to 1980, at which point the trend reversed (Figure 1). The rate of increase prior to 1980 cannot be reliably estimated as the historical data are too sparse, but the post‐1980 data obtained at the breeding sites show parallel decreases in the three regions at a common rate of −0.11 ± 0.02 mm/year, producing a decrease of 3.85 mm, or about 4%, between 1980 and 2015. The data from both migration sites with relevant data also show decreases of this magnitude between the 1980s and the 2010s. In contrast to wing lengths, measurements from the same individuals showed no substantial or consistent changes in bill lengths (Figure 1).

Differences in measurement technique among individual investigators are undoubtedly represented in our data, but given the large number of research groups and persons within groups measuring birds, improbably specific, sequential, and parallel biases would have had to have occurred to produce these general results. Flattened wing length measurement biases among individuals are on order of <1.0 mm (see Appendix), substantially less than the effect sizes reported here. Furthermore, most of the annual means we analyzed were themselves pooling of measurements made by multiple individuals whose individual biases would tend to offset one another. Finally, substantial change occurred within sites despite having consistent investigator training (e.g., Nome 1993–2014, and the Bay of Fundy in the 1980s, Table 1). A decrease in wing length at the Bay of Fundy during the 1980s, for example, occurred when all birds were measured by PH. Observer differences were more likely to add noise and obscure patterns in these data rather than create them. The results from Manomet, which were measured with a less repeatable wing chord methodology, may have been influenced by biases (see below).

Due to phenotypic covariance (Table 4), contemporaneous changes in wing and bill metrics could be driven to some extent, in principle, by overall changes in body size and/or correlated selection (Lande & Arnold, 1983). While some parallel patterns do occur in the short term, most strikingly visible in the consistently measured and large James Bay samples 1975–1982, the longer‐term patterns in most of our data sets differ between wings and bills. Independent change thus occurred at this timescale: wing lengths change in clear and consistent patterns, whereas bills show weak and/or geographically inconsistent patterns. Bill lengths were maintained in these data in general, from museum lengths through to the present, suggesting little change in factors affecting their size distributions, even in the face, potentially, of changes in wing sizes with common developmental bases. Thus, a general change in body size cannot account for the patterns of wing and bill changes; instead, body shapes as defined by at least these two metrics have diverged.

### 4.1 Interpreting changes at migratory sites

Processes other than true phenotypic change can complicate interpretation of the data from migratory stopover sites (see section 1). Hicklin and Chardine (2012) attributed changes in metrics observed at the Bay of Fundy between 1982 and 2006 to a reduction in the representation of birds from the eastern (i.e., larger‐bodied) breeding region, and inferred that eastern breeding populations had declined relative to other breeding areas. This inference was consistent with both wing and bill length data collected from 1982 to 1989, and with bill length data through 2006; however, due to the annual differences in wing measurement techniques 1997–2006 now recognized, no conclusions can be drawn from the large differences in wing lengths between time periods. A highly detailed analysis of bill lengths at the Bay of Fundy 1985–2015 argues against disproportionate changes in regional representation over the entire period, but offers no alternative explanation for the short bill sizes in the early 2000s (Bliss 2015).

Changes in wing lengths of breeding semipalmated sandpipers reported in the present study, unknown to Hicklin and Chardine (2012), combined with changes in the phenotypic covariance between wing and bills (Table 4), suggest a hypothesis that could account for both wing and bill length patterns in the Bay of Fundy data. The 0.56 mm bill size decline originally reported through 2006 and also captured in the quadratic relationships we show (Figure 1), could result from a genetic correlation at least partly driving the phenotypic correlation with wing lengths, as previously argued for the correlated changes in both metrics detected in the large samples at James Bay. The subsequent lengthening of bills since 2002 would then represent a lagging shift in the underlying genetic correlation in response to selection for maintaining historical bill lengths in the face of shorter wing lengths. The decreased slopes of bills on wings since 1980 shows that these metrics have changed nonallometrically (Table 4), and phenotypic covariances can be reasonable surrogates for genetic covariances (Agrawal & Stinchcombe, 2009).

The extensive data from Manomet, while noisy, probably due to shifting measurement biases, nonetheless present a contrasting situation from this perspective. In contrast to all other post‐1980 data, wing lengths increased, but bill lengths did not (Figure 1). This suggests true disproportionate use of the site by longer‐winged migrant sandpipers. A potential explanation for this is that since the late 1980s, longer‐winged (eastern) birds may have disproportionately avoided the Bay of Fundy in favor of utilizing sites further south, possibly due to the increasing numbers of introduced locally breeding peregrine falcons at Fundy (*Falco peregrinus*; Dekker, Dekker, Christie, & Ydenberg, 2011). Whether such a shift would be expected also depends on raptor population changes at other stopover sites (Ydenberg, Barrett, Lank, Xu, & Faber, 2017), but recent geolocator tracks of individual southward migrating semipalmated sandpipers from Coats Island show 8 of 12 passing south of Fundy (Brown et al., 2017).

### 4.2 Ecological causes of morphometric patterns

Any single hypothesis to explain the phenotypic changes documented here must account for (1) the pre‐1980 increase in wing length, (2) the subsequent decline, (3) the timing of the peak, and (4) different patterns occurring in wing and bill lengths. A first hypothesis, raised in other studies, is that the phenotypic changes are proximate responses to environmental change to which feather growth strategies of adult semipalmated sandpipers are lagging in time or have limited capacity to adapt. For example, despite selection favoring longer bill lengths, van Gils et al. (2016) attributed a substantial shortening of bill lengths in Red Knot populations as resulting from increasing phenological mismatch with resources during the critical period of chick growth (see also Husby et al., 2011). A similar process could operate here, if shrinking body size is a universal response to global temperature increase (Gardner, Heinsohn, & Joseph, 2009; Gardner et al., 2011, 2014). In the system described here, the lack of concordant changes in bill length suggests an effect specific to wing length *per se* rather than a generalized change in body size. More importantly, while the timing of the decline in wing length since 1980 broadly matches a global temperature increase, an increasing wing length prior to 1980 is opposite to what would be expected under this mechanism.

A second possible mechanism is that wing wear during migration and the nonbreeding season has increased, e.g., due to habitat or range changes (Fahrig, 2003). In the closely related and ecologically similar western sandpiper (*Calidris mauri*), adults lose 4.2–5.4 mm from their primaries during the nonbreeding period following molt (O'Hara, Fernández, Haase, de la Cueva, & Lank, 2006). Assuming that semipalmated sandpipers have similar ecology, the total wear would have had to increase by 70%–90% since 1980 to account for an additional 3.85‐mm loss. If most wear occurs during flight, additional wear of this magnitude appears unrealistic and, as with phenotypic mismatch, this hypothesis requires a reversal of selective direction around 1980 to account for the patterns presented. While not impossible, no such reversal has yet been identified.

A third possibility is that the changes in wing size reflect changes in selection on performance attributes of wing size and shape that affect the balance between the fitness consequences of energetic flight efficiency versus agility (Swaddle & Lockwood, 1998). Flattened wing length of small sandpipers correlates strongly with principle component indices of wing shape derived from measurements of all 10 primaries; longer wings of semipalmated sandpipers are more pointed and shorter wings more rounded (Fernández & Lank, 2007; Ortiz et al., unpublished). Several factors could alter this balance.

Longer wings improve long‐distance flight efficiency, and even small changes can be important. The effect on migratory performance of a ~4% reduction in wing length can be approximated by comparing the calculated flight performance of male and female western sandpipers (E Rowland & RCY, unpublished, using Program Flight version 1.14; Pennycuick, 2008). Females are larger and have about 25% more range: at 25 g, for example, a female had a range of 1884 km compared to a male's range of 1503 km. The exact value depends on many parameters, but wing shortening of even a few millimeters can be expected to have a measurable and negative impact on migratory flight efficiency and range of small sandpipers. Despite potential costs of this magnitude, wings have become shorter.

Greater agility in shorebirds can also be favored by sexual selection for aerial displays, (Figuerola, 1999; Székely, Reynolds, & Figuerola, 2000), which would operate primarily on males. Here we are considering mixed sex populations, and there is no reason to believe that display modality is shifting toward greater aerial display or that there were changes in the intensity of competition among males. If anything, recently reported population declines of semipalmated sandpipers (Andres et al., 2012) might reduce population densities and therefore intrasexual competition on breeding grounds, which would not favor shorter wing lengths.

Wing shapes of small shorebirds may be influenced by human hunting, which has become a conservation concern for several species in the Western Hemisphere. Along the coasts of Guyana, French Guiana, and Suriname, long the core historical wintering range for semipalmated sandpipers, trembling or “choking wires” stretched from posts across beaches have long been used to knock low‐flying birds out of the air, but mist nets are now also widely used (Ottema & Spaans, 2008; Morrison et al., 2012). Both techniques would select against less agile birds and thus favor shortened wing lengths. We lack information on historical shifts in hunting techniques or intensity (Watts & Turrin, 2016), thus we cannot easily try to match the timing of the effect of human harvest on the wing length changes we have documented.

We suggest that historical changes in avian predator abundance provide the most consistent and parsimonious explanation for the wing size pattern we have documented. Avian raptors can be responsible for substantial mortality of local overwintering shorebirds (e.g., 5%–14% of a local population by a single merlin: Page & Whitacre, 1975) and can account for the annual local mortality of 20%–60% of juveniles (Whitfield, 2003). In addition to this mortality, there may be trait‐mediated effects if individuals with longer wings pay a cost for behaving more cautiously, such as a lower foraging intake rate (McNamara & Houston, 1987). Major avian predators of small sandpipers, such as peregrine falcons and merlins (*F. columbarius*; Page & Whitacre, 1975; Dekker et al., 2011) experienced severe hemispheric‐wide DDT‐induced population decreases during the late 1940s–1970s, but have undergone a continuing and strong population recovery as DDT was banned for widespread use in most of North America in 1973 (Cade, Enderson, Thelander, & White, 1988; Dekker et al., 2011; Brandes, Oleyar, Hoffman, & Goodrich, 2013; Ydenberg et al., 2017). Small shorebirds have a well‐documented diverse range of behavioral tactics and body mass sensitivity in response to changes in predation danger at different time and spatial scales (e.g., Hilton, Ruxton, & Cresswell, 1999; Lank, Butler, Ireland, & Ydenberg, 2003; Ydenberg et al., 2004, 2010; Pomeroy, Butler, & Ydenberg, 2006; Sprague, Hamilton, & Diamond, 2008; Beauchamp, 2010; Fernández and Lank, 2010; Martins et al., 2015, and references therein). We hypothesize that parallel changes have also occurred in morphology. Under this hypothesis, the peak wing length value observed ca. 1980 represents the end of a period during which low predation danger allowed the benefits of flight efficiency for migration to select for longer wings. When falcon numbers rebounded, the balance of selection reversed, increasingly favoring more defensive (shorter and rounder) wing morphology.

We estimate the post‐1980 rate of phenotypic decline in wing length at 1,098 darwins and 0.176 haldanes, which fall well within the range estimated by other studies of microevolutionary change of a decade or longer in duration (see Table 1 in Hendry & Kinnison, 1999; Kopp & Matuszewski, 2013). Hence, it is plausible that these changes are primarily a direct genetically based response to selection. In addition to or along with selection, the change in size could be an induced defense, as in crucian carp (*Carassius carassius*), in which a deeper body form is induced by the presence of predatory pike (*Esox lucius;* Domenici, Turesson, Brodersen, & Bronmark, 2008). The altered body form of carp lowers vulnerability and enables higher speed, acceleration, and turning rate during anti‐predator responses. It is in theory possible that individual semipalmated sandpipers alter feather morphology during the annual wing molt based on experience gained during the previous southward migration or a more generalized assessment of their danger landscape. Such a mechanism not been demonstrated in birds, to our knowledge, although wing morphology commonly changes toward higher performance (longer) wings between juvenile and adult phases of avian species (Alatalo, Gustafsson, & Lundbürg, 1984; Fernández & Lank, 2007). A further possibility is that adjustments to wing morphology result from a maternal effect in which the mother's assessment of predation danger leads to an adjustment of egg contents that alter her offspring's phenotype. As a current avian example, experimentally increasing levels of perceived predation risk increased yolk testosterone in clutches of great tits, and young hatching from treated nests grew wings at faster rates and had longer wings at maturity than those in control nests (Coslovsky & Richner, 2011; Coslovsky, Groothuis, de Vries, & Richner, 2012).

## 5 Conclusion

The wing lengths of semipalmated sandpiper populations increased during the decades prior to 1980 and subsequently decreased with no accompanying systematic changes in bill length. The changes in wing length documented here coincide temporally with continent‐wide changes in predation danger attributable to the steep decline and subsequent ongoing recovery of falcon populations resulting from the postwar introduction of DDT and 1973 ban on its widespread agricultural use. We hypothesize that the ongoing changes in wing size reflect changes in the balance of selective pressures arising from efficient long‐distance migration (longer, more tapered wings) versus defensive morphology (shorter, rounder wings). Several alternative hypotheses, including that a general body size reduction is a universal response to climate warming, do not account for the initial increase in wing lengths and/or the lack of systematic changes in bill size. If the predation danger hypothesis is true, it should apply more generally to small shorebirds and other avian taxa (Yom‐Tov et al., 2006). We encourage continued exploration of historical changes during the pre‐ and post‐DDT time frame to challenge this hypothesis.

## Conflict of Interest

None declared.

## Figures and Tables

**Figure A1:**
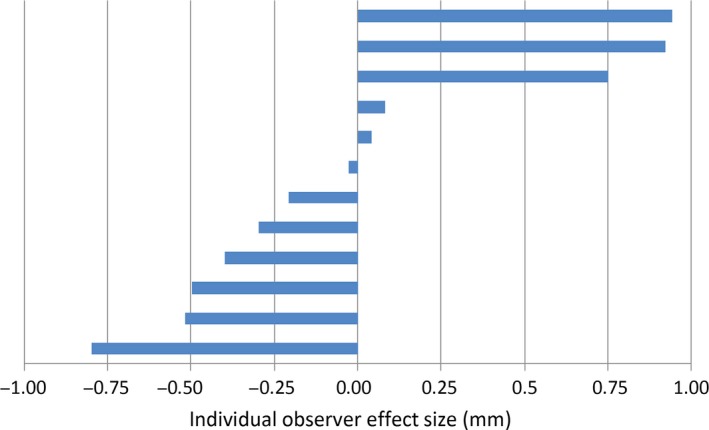
Estimated measurement biases of 12 individuals on flattened wing lengths, in mm, expressed as deviations from a mean of 101.2 mm. All individuals measured >350 wings during one year at James Bay 1974–1982; some did so in multiple years. Each bar is the estimate of individual observer effect from a maximum likelihood model (SAS, Proc mixed) predicting wing length as a function of individual (random effect, all *p* > .0001), year (*p* ranged from >.0001 to 0.54 among years), date measured (*p* = .112), and a year*date interaction term (*p* ranged from .003 to .763; without the interaction term *p* for date <.0001)

## Appendix 1

### Estimating Interobserver Variability in Wing Length Measurements

#### 1

We present three comparisons to evaluate interobsever variability in wing length measurements. First, at James Bay 1975–1982, 12 different observers measured a total 20,135 semipalmated sandpiper wing lengths (368–2,452 birds /observer/year). Figure A1 plots the modeled deviation of each observer from the global mean. Seven of the 12 observers fall within 0.5 mm of the global mean. The mean difference between the 66 possible pairs of observers was 0.66 ± 0.46 *SD* mm, with a maximum difference of ~1.7 mm between individuals.

Ideally, estimates of interobserver variability should be based on measurements of the same bird. The James Bay data included 35 recaptures each measured independently within a season by somewhat random pairs of two of eight observers. We estimated the repeatability (intraclass correlation coefficient Lessells & Boag, 1987) of these measurements as 0.34. Based on an observer variance component of 0.72, 67% of observations between observers should fall within 0.85 mm (±1 *SD*) of the mean value, and 95% of values within 1.70 mm (±2 *SD*). Ten of the 35 measurements were identical; but the actual mean difference between measurements was more substantial than in the larger population comparisons, being 2.0 ± 1.76 (*SD*) mm, or that determined for western sandpipers in a third comparison. Sixteen western sandpipers, a simlarly sized close relative of semipalmated sandpipers, were measured in a blinded procedure by each of five observers at Roberts Bank, British Columbia, in August 2014. The repeatibility was 0.62, and the observer variance component was 0.52; thus, 67% of observations between observers should fall within 0.72 mm (±1 *SD*) of the mean, and 95% of values within 1.44 mm (±2 *SD*). The actual mean difference between the 10 possible pairs of observers was 0.97 ± 0.65 (*SD*) mm.

We conclude that individual wing length measurement baises among observers involved in this study are on the order of ± <1.0 mm from a global mean.
